# 

**Published:** 1893-10-14

**Authors:** 


					HOSPITAL, October 14th,
L PRICE TWOPENCE. If ,nwith extra nu?rsinq supplemen1
T-W E - cswrssK-
? "^namiaaion in the United Kingdom and Abroad,] ^ Ditto per Annum, 8s., post free.
No. 368. Vol. XV. Saturdav,
October 14th, 1893.
[Twopence.

				

